# Iron-Deficiency Anemia Results in Transcriptional and Metabolic Remodeling in the Heart Toward a Glycolytic Phenotype

**DOI:** 10.3389/fcvm.2020.616920

**Published:** 2021-01-21

**Authors:** Yu Jin Chung, Pawel Swietach, M. Kate Curtis, Vicky Ball, Peter A. Robbins, Samira Lakhal-Littleton

**Affiliations:** ^1^Department of Physiology, Anatomy and Genetics, University of Oxford, Oxford, United Kingdom; ^2^The Rayne Institute, St Thomas' Hospital, London, United Kingdom

**Keywords:** iron deficiency, anemia, hypoxia, RNA-Seq, cardiac transcriptome, cardiac metabolism

## Abstract

Iron deficiency is the most prevalent micronutrient disorder globally. When severe, iron deficiency leads to anemia, which can be deleterious to cardiac function. Given the central role of iron and oxygen in cardiac biology, multiple pathways are expected to be altered in iron-deficiency anemia, and identifying these requires an unbiased approach. To investigate these changes, gene expression and metabolism were studied in mice weaned onto an iron-deficient diet for 6 weeks. Whole-exome transcriptomics (RNAseq) identified over 1,500 differentially expressed genes (DEGs), of which 22% were upregulated and 78% were downregulated in the iron-deficient group, relative to control animals on an iron-adjusted diet. The major biological pathways affected were oxidative phosphorylation and pyruvate metabolism, as well as cardiac contraction and responses related to environmental stress. Cardiac metabolism was studied functionally using *in vitro* and *in vivo* methodologies. Spectrometric measurement of the activity of the four electron transport chain complexes in total cardiac lysates showed that the activities of Complexes I and IV were reduced in the hearts of iron-deficient animals. Pyruvate metabolism was assessed *in vivo* using hyperpolarized ^13^C magnetic resonance spectroscopy (MRS) of hyperpolarized pyruvate. Hearts from iron-deficient and anemic animals showed significantly decreased flux through pyruvate dehydrogenase and increased lactic acid production, consistent with tissue hypoxia and induction of genes coding for glycolytic enzymes and H^+^-monocarboxylate transport-4. Our results show that iron-deficiency anemia results in a metabolic remodeling toward a glycolytic, lactic acid-producing phenotype, a hallmark of hypoxia.

## Introduction

Iron deficiency is a common comorbidity in chronic heart failure and is demonstrably associated with higher mortality rates (1–3). When induced in laboratory animals, it can result in fatal cardiac derangements (4–6). Severe iron deficiency leads to anemia, and there is growing evidence that impaired oxygen transport contributes to cardiac disease and death (7–9). Given the myriad of biological functions related to iron and oxygen, the cardiac consequences of iron-deficiency anemia (IDA) are inherently complex. Metabolism is a likely target of remodeling (10) because the heart normally engages in a high rate of aerobic respiration, largely of fatty acids (11, 12). In anemia, inadequate convective O_2_ transport by blood and dysfunctional O_2_ handling in cardiac myocytes is predicted to shift metabolism toward a more glycolytic phenotype as an adaptation to a more limited oxygen supply. An elevated glycolytic rate will also lead to an enhanced production of lactic acid (13), potentially reducing intracellular pH (pH_i_), a major modulator of cardiac function (14, 15). In addition to a metabolic effect, iron-deficiency anemia may alter cardiac gene expression through oxygen- and iron-sensitive enzymes such as prolyl hydroxylases (PHDs) and lysine demethylases (KDMs), which regulate the transcription factor hypoxia inducible factor (HIF) (16, 17) and histone methylation mark (18–20), respectively. To gain unbiased insight into the possible changes in cardiac function under IDA, a non-candidate approach is warranted.

Here, we sequenced the transcriptome of cardiac muscle of mice, rendered iron deficient by restricting their dietary iron intake. We find that IDA resulted in marked differences in transcriptomic profile compared to hearts from mice on a control diet. Differentially expressed genes related to processes such as mitochondrial metabolism, stress response, immune system, and various signaling processes, including calcium handling. To verify that the metabolic profile is, indeed, affected by IDA, further studies used a combination of *in vitro* and *in vivo* methods to assess oxidative mitochondrial pathways and pyruvate metabolism. We confirm that iron-deficiency anemia produces a marked shift toward lactic acid producing glycolytic metabolism.

## Materials and Methods

### Animal Procedures

Animal procedures were performed in compliance with Home Office Guidance on the Operation of the Animals (Scientific Procedures) Act of 1986 and the University of Oxford institutional guidelines. All treatments administered to animals were approved by the Home Office under the Project License 30/3182. Male wild-type C57BL/6J mice used in this study were housed in individually ventilated cages, with a minimum of 2 and maximum of 6 mice per cage. At the end of the protocol, animals were killed humanely by cervical dislocation. Hearts were washed in ice-cold PBS and snap frozen in liquid nitrogen.

### Iron Deficiency Model

At 3 weeks of age, mice were weaned on either an iron-deficient diet (2–5 ppm iron; Teklad, TK99397; Envigo) or an iron-adjusted diet (200 ppm iron, Teklad, TK08713; Envigo) for 6 weeks. Hemoglobin concentration was measured from tail-vein blood, collected using a 27G needle and a HemoCue device (Radiometer).

### Dynamic Nuclear Polarization (DNP)-Hyperpolarised-^13^C_1_ Magnetic Resonance Spectroscopy

Scans were performed between 7 a.m. and 1 p.m, during the early absorptive state. Anesthesia was induced at 3% isoflurane and maintained at 2%. A catheter with a 32 G needle was inserted into the tail vein for intravenous infusion of hyperpolarized solution. The animal was placed into a home-built cradle and body temperature maintained via a warm air flow through a heat-exchanger placed on either side of the animal. A circular custom-built ^13^C RF surface transmit/receive coil with a 10 mm radius was placed on the chest over the heart and secured with surgical tape. ECG probes were inserted into each forepaw and monitored throughout the experiment.

MR spectroscopy was performed using a 7 T (300 MHz) horizontal bore MR scanner interfaced to a direct-drive console (Varian Inc). Using a 72 mm ^1^H volume transmit/receive RF coil (Rapid Biomedical), a 3-plane FLASH localizer image was obtained to confirm the location of the heart at the magnet isocenter. Following dissolution, 150 μL of hyperpolarized pyruvate was injected over 10 sec into the mouse, followed by a 50 μL flush of heparinized saline. Sixty individual ECG-gated ^13^C MR pulse-acquire cardiac spectra were acquired over 2 min following injection. The frequency was centered on the C_1_ pyruvate resonance.

^13^C spectra were analyzed using the AMARES algorithm in the jMRUI software package. Spectra were DC offset-corrected based on the last half of acquired points. A kinetic model was obtained by quantification of the peak areas of [1-^13^C]pyruvate, [1-^13^C]lactate and [1-^13^C]bicarbonate at each time point and normalized to the peak area of [1-^13^C]pyruvate. A summed model was obtained by adding the averaged maximum peak area of each metabolite over 30 individual spectra from the first appearance of pyruvate and normalized to the peak area of [1-^13^C]pyruvate.

### Pyruvate Polarization and Dissolution

Approximately 40 mg of [1-^13^C]pyruvic acid doped with 15 mM trityl radical (OXO63, Oxford Instruments) and 3 μL Doatrem (1:50 dilution, Guebert) was hyperpolarized in a General Electric Prototype Polariser (GE Healthcare) with 45 min of microwave irradiation. The sample was then dissolved for 20 min in a pressurized and heated alkaline solution, containing 60 mM sodium hydroxide and 247 mM EDTA dipotassium salt (Sigma). This yielded an 80 mM solution of hyperpolarized sodium [1-^13^C]pyruvate with a polarization of ~30% at physiological pH and temperature.

### Isolation of Cardiac Myocytes

Excised hearts were cannulated at the aorta using a blunted 23 G needle, mounted on a Langendorff apparatus. Hearts were perfused with warm Tyrode perfusion solution containing (in mM): 130 NaCl, 5.6 KCl, 3.5 MgCl_2_, 5 HEPES, 0.4 Na_2_HPO_4_, 10 Glucose, and 20 Taurine, pH 7.4 at 37°C, then digested with Tyrode perfusion solution supplemented with 0.1 mM CaCl_2_ and 1.0 mM liberase (Sigma). Following digestion, cells were dissociated by careful mechanical disruption and filtered through a 500 μm cell strainer. Enzymatic activity was quenched by adding 1% BSA in Tyrode perfusion buffer. CaCl_2_ was reintroduced step-wise to a final concentration of 1 mM prior to use.

### RNA and DNA Extraction

Total RNA was extracted from ~30 mg of snap frozen, crushed cardiac tissue using the RNeasy Fibrous Mini Kit (Qiagen), following manufacturer's instructions. Genomic DNA (gDNA) was extracted from ~30 mg of snap frozen, crushed cardiac tissue using the DNeasy Blood, and Tissue Kit (Qiagen), following manufacturer's instructions.

### Sequencing

For library preparation, mRNA was selected from total RNA by a poly-(A) selection before conversion to cDNA and adapter-ligated. Samples were sequenced on the Illumina HiSeq 4000. Samples with RNA integrity number of at least 6 were included in this analysis. A total of 20 samples were submitted for sequencing across 2 units, each of which received approximately 240 million pair-ended reads. Raw reads were 75 base-pairs in length. The analysis pipeline involved using Spliced Transcripts Alignment to a Reference (STAR) (21) to align raw reads to the mouse reference genome *mm10*. PCR duplicates were removed using the sequence alignment/map (SAM) tools (22) and counts table generated using featureCounts (23). Differential gene expression analysis was conducted in RStudio using DESeq2 (24) and EdgeR (25) for genes with adequate expression (at least one count per million), after correcting for PCR duplicates and normalizing to library size. Pathway analysis was conducted using the Generally Applicable Gene Set Enrichment (GAGE) (26) and pathway map was generated using PathView (27). One sample from each group was excluded due to sample contamination. In some instances, gene expression level between groups for a particular gene of interest was compared using mapped reads generated by featureCounts using RNA-seq data, and are presented in counts per million (cpm).

### Quantitative PCR

cDNA was reverse transcribed from total RNA using the Expand Reverse Transcriptase kit (Roche) and poly dT primers (Invitrogen), following manufacturer's instructions. For qPCR, 25 μg of cDNA was used as template and either the Taqman Fast Universal or PowerUp SYBR Green master mix (Thermo Fisher). *Gapdh* (SYBR green assays) or *Actb* (Taqman assays) was used as a housekeeper. Primers used for Taqman assays are: *Pdk1* (Mm00554300), *Pdk4* (Mm01166879), and *Ldha* (mM01612132). Primer used for SYBR green assays are:

*Cox4-1*: 5′ CAGGCTTCCGTCTTAACCGTTG; 3′ TCAGCGTAAGTGGGGAAAGCA

*Cox4-2*: 5′ GTTGACTGCTACGCCCAGC; 3′ AGGCCACCTTCTCTGCTTGG

*Gapdh*: 5′ GGCACAGTCAAGGCTGAGAATG; 3′ ATGGTGGTGAAGACGCCCAGTA.

### Mitochondrial Copy Number Assay

Mitochondrial (mt) copy number was determined as a ratio of mtDNA:gDNA. The mt primer is located within the *Nd4* gene in the mitochondrial genome, which encodes for the NADH dehydrogenase 4 protein: 5′ CCAACTACGAACGGATCCACA; 3′ TGATTGAAGGGGGTAGAGCTAGA. The genomic primer was designed to span the exon-intron junction of the β-actin genomic sequence: 5′ TAGGGTCCGGGCCTCGAT; 3′ TGTCTCGGTTACTAGGCCTGC. qPCR was carried out in PowerUp SYBR Green Master Mix with 20 ng gDNA template.

### Immunofluorescence

Isolated cardiomyocytes were plated on to laminin coated plates and fixed in ice-cold 4% paraformaldehyde. Cells were permeabilized in 0.3% triton-X and blocked in primary antibody overnight at 4°C, then in secondary antibody and DAPI for nuclear staining. Cells were imaged on a Zeiss LSM 700 confocal microscope at 40x magnification.

### Transmission Electron Microscopy (TEM)

Isolated cardiac myocytes were fixed in 2.5% glutaraldehyde and stored in 0.25% glutaraldehyde at 4°C. Samples were rinsed 5 times in 1X PBS, 15 min each wash, then fixed in 1% osmium tetroxide (OsO_4_; TAAB Laboratories) in 1X PBS at 4°C for 2 hrs with gentle rotation. Samples were rinsed 5 times in ddH_2_O, 15 min each wash, and fixed in 0.2% uranyl acetate (Agar Scientific) overnight at 4°C in the dark. Samples were washed once in ddH_2_O for 10 min. For dehydration of specimen, samples were washed for 15 min at 4°C, in 30, 50, 70, 80, 90, and 95% EtOH. Samples were then incubated 3 times in 100% dry EtOH at 4°C, 30 min each incubation step. For resin infiltration, samples were incubated in 2:1 100% dry EtOH:TAAB TLV resin (TAAB Laboratories) for 2 hrs, 1:1 100% dry EtOH:TAAB TLV resin for 3 hrs, and 1:2 100% dry EtOH:TAAB TLV resin for 2 hrs with gentle rotation. Samples were then incubated in 100% TAAB TLV resin for 48 hrs at room temperature. During this time, the resin was changed every 8 hrs. Tissue pieces were transferred to Beem capsules filled with fresh 100% TAAB TLV resin and polymerized overnight at 60°C. Samples were sectioned on the Leica UC7 ultramicrotome. Resin blocks were faced-up using a glass knife in preparation for ultra-thin sectioning. For ultra-thin sectioning, 90 nm thin sections were cut using a diamond knife (Diatome) and transferred to a 200 mesh Cu grid (TAAB Laboratories). Prior to imaging, grids were post-stained with Reynold's lead citrate for 5 min and washed 3 times in ddH_2_O and air-dried overnight. TEM imaging was performed at 120 kV on the FEI Tecnai 12 and images acquired using the Gatan OneView CMOS camera with Digital Micrograph 3.0 software.

### ETC Complex Assays

Cardiac homogenates were prepared using approximately 10 mg of frozen, crushed tissues suspended in 200 μL of ice-cold KME buffer (in mM): 100.0 KCl, 50.0 Mops, 0.5 EGTA (pH 7.4 at room temperature with NaOH). Samples were homogenized by rupturing with a TissueRuptor (Qiagen) over ice and used immediately to measure the activities of mitochondrial complexes.

Complex I activity was measured as the rate of NADH oxidation at 340 nm and 30°C using a spectrophotometer. The assay buffer contained the following (in mM, unless otherwise specified): 25 potassium phosphate (pH 7.2 at room temperature), 5 MgCl_2_, 0.13 NADH (MP Biomedicals, LLP), 3.65 antimycin A (Santa Cruz), 65 μM coenzyme Q1 (Sigma), and 250 mg fatty acid free BSA (Sigma). The reaction was carried out in 1 mL assay buffer supplemented with 20 μL of protein lysate and read for 1 min at 340 nm against a blank containing ddH_2_O. As a negative control, 200 μM of rotenone (Sigma) was added to a new reaction and the inhibited rate measured for 1 min. The activity of Complex I was determined by dividing the gradient of the absorbance change over the extinction coefficient (6810 nmol·min^−1^) and expressed in nmol·min^−1^·mg protein^−1^.

Complex IV activity was measured as the rate of oxidation of cytochrome c^2+^ at 550 nm and 30°C using a spectrophotometer. The assay buffer contained 10 mM potassium phosphate pH 7.0 and 0.5 μM reduced cytochrome c. The reaction was carried out in 1 mL assay buffer supplemented with 5 μL protein lysate and read for 3 min at 550 nm against a blank of assay buffer supplemented by 100 μM potassium ferricyanide. As a negative control, 10 μM of sodium azide was added to a new reaction and the inhibited rate measure for 3 min. The first-order rate constant (*k*) was calculated as previously described (28). Briefly, the natural logarithm was taken for the absorbance at the time points *t* = 0, 60, 120, 180 sec and the difference for each pair of time points determined [t(60)-t(0), t(120)-t(60), t(180)-t(120)]. The average of these differences was taken to be *k* and the activity expressed in *k*·min^−1^·mg protein^−1^.

Reduced cytochrome c^2+^ was prepared in a Visking 7000/1 dialysis tubing hydrated for 30 min in 1 L ddH_2_O supplemented with 20 g sodium carbonate and 0.372 g EDTA at 80°C. A solution of 100 mg of cytochrome c from bovine heart and 10 mg sodium ascorbate dissolved in 10 mL potassium phosphate (0.1 M, pH 7.0 at room temperature) was added to the tubing and dialysed against 1 L of 0.1 M potassium phosphate buffer for 24 hrs at 4°C. The phosphate buffer was exchanged 3 times, every 8 hrs. The redox state of the synthesized reduced cytochrome c was verified by measuring the absorbance spectra between wavelengths of 500 and 600 nM, in the presence or absence of 100 μM potassium ferricyanide (Sigma) and compared against the oxidized cytochrome c.

### Western Blots

Cardiac lysate was prepared from crushed, frozen heart tissue using RIPA buffer supplemented with complete protease inhibitor. For SDS-PAGE, 100 μg of protein lysate was loaded. Membrane was blocked in primary antibody overnight at 4°C, then in HRP-conjugated secondary antibody.

### Antibodies

rb-HIF1α NB100-479 (Novus Biological), rb-COX4-1 NB110-39115 (Novus Biological), ms-COX4-2 H00084701-M01 (Novus Biological), rb-α-tubulin ab4074 (Abcam), rb-H3 ab1791 (Abcam), rb anti-ms MTP1 (FPN) MTP11-A (alpha-Diagnostics Inc.), gt-anti-rb-HRP sc-2030 (Santa Cruz), gt-anti-ms-HRP sc-2031 (Santa Cruz), dk anti-rb AlexaFluor 488 Ab150073 (Abcam).

### Statistics

Data are reported as mean ± SEM. For RNA-seq analysis, the default statistical test for each Bioconductor package was used. For all other assays, two-tail, unpaired student's *t*-test was used to compare iron-deficient group mean with the control group. ^*^p < 0.05, ^**^p < 0.01, ^***^p < 0.001, ^****^p < 0.0001. Number of animals (n) used per experiment is in the figure legend.

## Results

### Iron-Deficient Diet Depletes Iron Stores and Produces Severe Anemia in Mice

**Figure 1:**
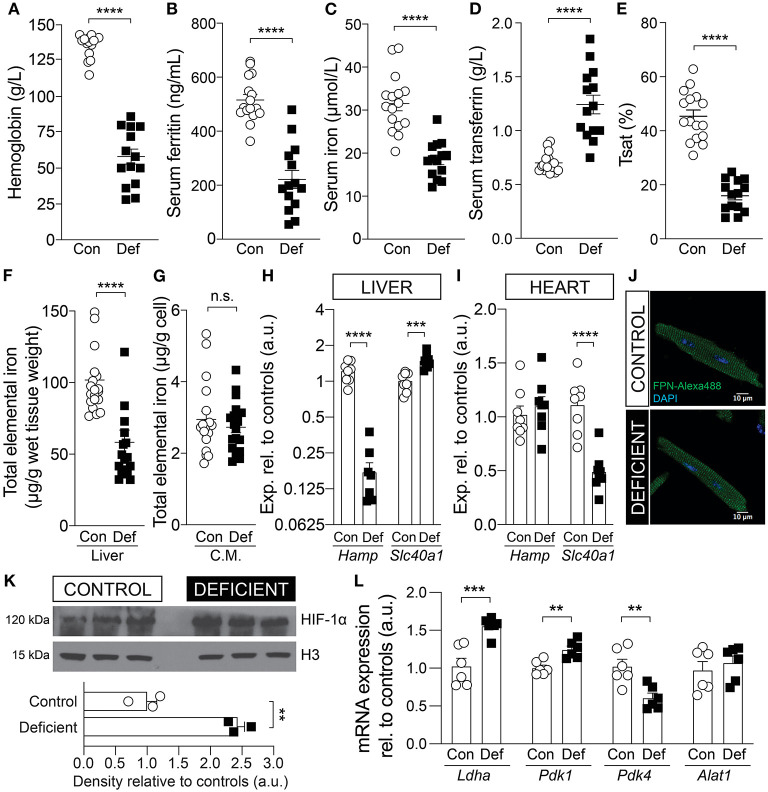
Validation of iron status in mice following 6 weeks of an iron-deficient or control diet. Measurement of **(A)** blood hemoglobin, **(B)** serum ferritin, **(C)** serum iron, **(D)** serum transferrin, and **(E)** calculated transferrin saturation (Tsat). *n* = 14 mice per group. Total elemental iron in **(F)** liver and **(G)** isolated cardiac myocytes (C.M.) measured by ICP-MS. *n* = 20 mice per group. Comparing the mRNA expression level of hepcidin (*Hamp*) and ferroportin (*Slc40a1*) in the **(H)** liver and **(I)** whole heart tissue. *n* = 8 mice per group. **(J)** Immunofluorescence staining for ferroportin (FPN) protein in permeabilized myocytes. Exemplar images shown from *n* = 3 mice per group. **(K)** Immunoblot for HIF-1α protein expression in the hearts of control and iron-deficient mice. Nuclear loading control histone H3. *n* = 3 mice per group. **(L)** mRNA expression of cardiac *Ldha, Pdk1, Pdk4*, and *Alat1*, relative to controls. Control (Con) and Iron-deficient (Def). *n* = 6 mice per group. All values plotted as mean ± sem. *P-*values determined by unpaired, two-tail student's *t*-test. **p < 0.01, ***p < 0.001, ****p < 0.0001; n.s., not significant.

Mice were weaned at 3 weeks of age on an iron-deficient diet containing 2–5 ppm iron. Age-matched controls received an iron-adjusted diet (control; 200 ppm iron). Following 6 weeks of dietary intervention, hemoglobin levels, measured from tail-vein blood, was significantly lowered to 57 g/L in response to reduced dietary iron (Figure 1A). For confirmation of iron deficiency, animals were culled following 6 weeks of diet and assessed for body iron reservoirs. Serum ferritin, serum iron and transferrin saturation were reduced by 57, 41, and 65%, respectively, and total serum transferrin was raised by 77% in animals on an iron-deficient diet (Figures 1B–E). Dietary iron restriction also reduced total elemental iron content in the liver by 43% (Figure 1F) but not in the heart, as determined from measurements in isolated cardiomyocytes (Figure 1G). Furthermore, consistent with previously reported response to iron deficiency (29), the hepcidin mRNA, *Hamp*, was significantly reduced and the ferroportin mRNA, *Slc40a1*, was significantly elevated in the livers of iron-deficient mice compared to controls (Figure 1H). In the hearts of iron-deficient mice, *Hamp* expression remained unaltered but *Slc40a1* expression was significantly downregulated at the transcript level (Figure 1I). However, the level of the protein product of Slc40a1 (FPN) was unaltered (Figure 1J). The low blood hemoglobin indicates that animals had developed anemia in addition to iron deficiency, and therefore reduced O_2_ delivery to aerobic tissues, such as the heart.

### Iron-Deficiency Anemia Leads to Upregulation of the Hypoxia Inducible Factor HIF-1α

Diminished convective transport of oxygen results in tissue hypoxia (30). Hypoxia affects the activity of the oxygen sensitive PHDs, which regulate the stability of the transcription factor HIF-1α, a key mediator of cellular response to hypoxia. To confirm that IDA affects the heart, the cardiac tissue was assessed for markers of hypoxia. The cardiac lysates of iron-deficient and anemic mice (IDA mice) showed elevated HIF-1α expression (Figure 1H) and upregulated HIF-1α target genes lactate dehydrogenase (*Ldha*) and pyruvate dehydrogenase kinase 1 (*Pdk1*), but not alaminotransferase (*Alat*), a HIF-independent gene (Figure 1I). Pyruvate dehydrogenase kinase 4 *(Pdk4)*, a target of PPARα, which is down-regulated by HIF-1α (31), was down-regulated, further indicative of HIF-1α induction.

PHDs also use iron as a cofactor for enzymatic activity, thus HIF-1α can be induced by iron deficiency independent of oxygen status (32, 33). Evidence for cellular iron deficiency can be obtained from studies of transcripts containing the iron response element (IRE). The mRNA of the transferrin receptor (*Tfrc*) and the divalent metal transporter-1 (DMT1), known as *Slc11a2*, contain an IRE in their 3′ untranslated region and are upregulated in iron deficiency. In the hearts of IDA mice, *Tfrc* expression was significantly upregulated compared to controls (Supplementary Figure 1A), but *Slc11a2* was unchanged (Supplementary Figure 1B). Additionally, total iron content in cardiac cells was not affected in IDA mice (Figure 1G). The lack of coordinated changes in iron-regulated genes and unaltered iron level in isolated cardiomyocytes suggest that the induction of HIF-1α in this mouse model is more likely to be related to impaired oxygen supply due to anemia than to cardiac iron deficiency.

### Iron-Deficiency Anemia Has Profound Effects on Cardiac Gene Expression

**Figure 2:**
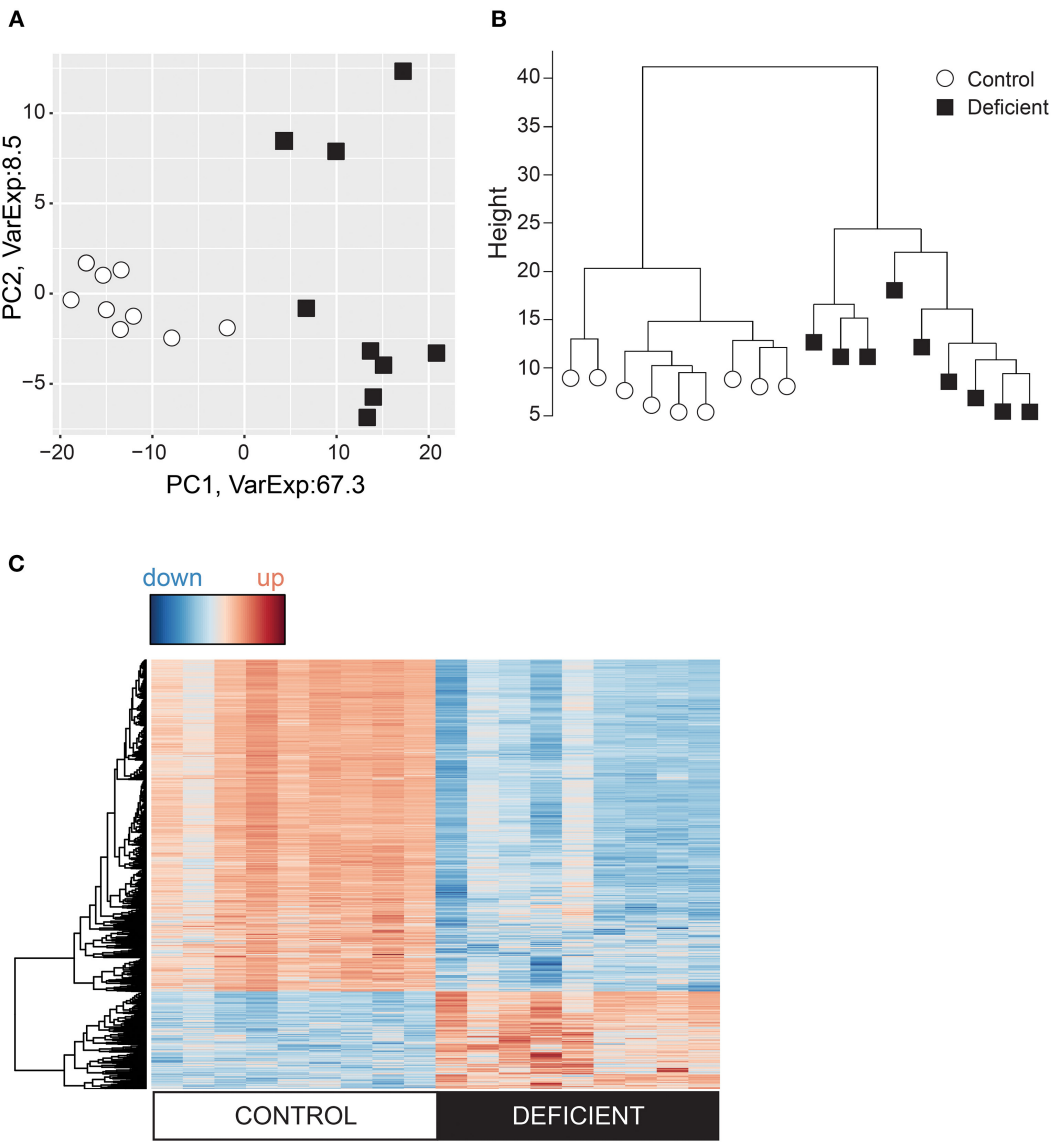
Analysis of the cardiac transcriptome using RNA-sequencing. Variation in the cardiac genes expression visualized in **(A)** Principal component analysis (PCA) and **(B)** Cluster dendrogram. **(C)** Heat map of differentially expressed genes where red = upregulated and blue = downregulated. *n* = 9 mice per group.

Tissue hypoxia can also affect chromatin structure and transcriptional regulation by modulating the activity of the oxygen sensitive histone demethylases KDMs (34, 35). Changes in histone methylation states have been previously observed in hypoxia (36, 37) and in anemia (38). To better understand how iron-deficiency anemia affects gene expression in the heart, the cardiac transcriptome was investigated by RNA-sequencing (RNAseq) of mRNA collected from whole hearts of mice following 6 weeks of iron-deficient or control diet. Differential expression analysis was performed on genes with adequate expression, corrected for PCR duplicates and normalized to gene size. Principle component analysis (Figure 2A) and the cluster dendrogram (Figure 2B) demonstrate a clear demarcation in gene expression pattern between IDA and control groups.

Differential gene expression analysis was carried out using EdgeR and DESeq2 packages and genes were considered to be differentially regulated if both packages identified these to have a *p* < 0.01 between IDA and control groups. According to these criteria, 1,569 differentially expressed (DE) genes were identified, of which 78% were downregulated in the IDA group (Figure 2C).

### Analysis of Differentially Expressed Genes: Gene Ontology and Pathway Analysis

**Figure 3:**
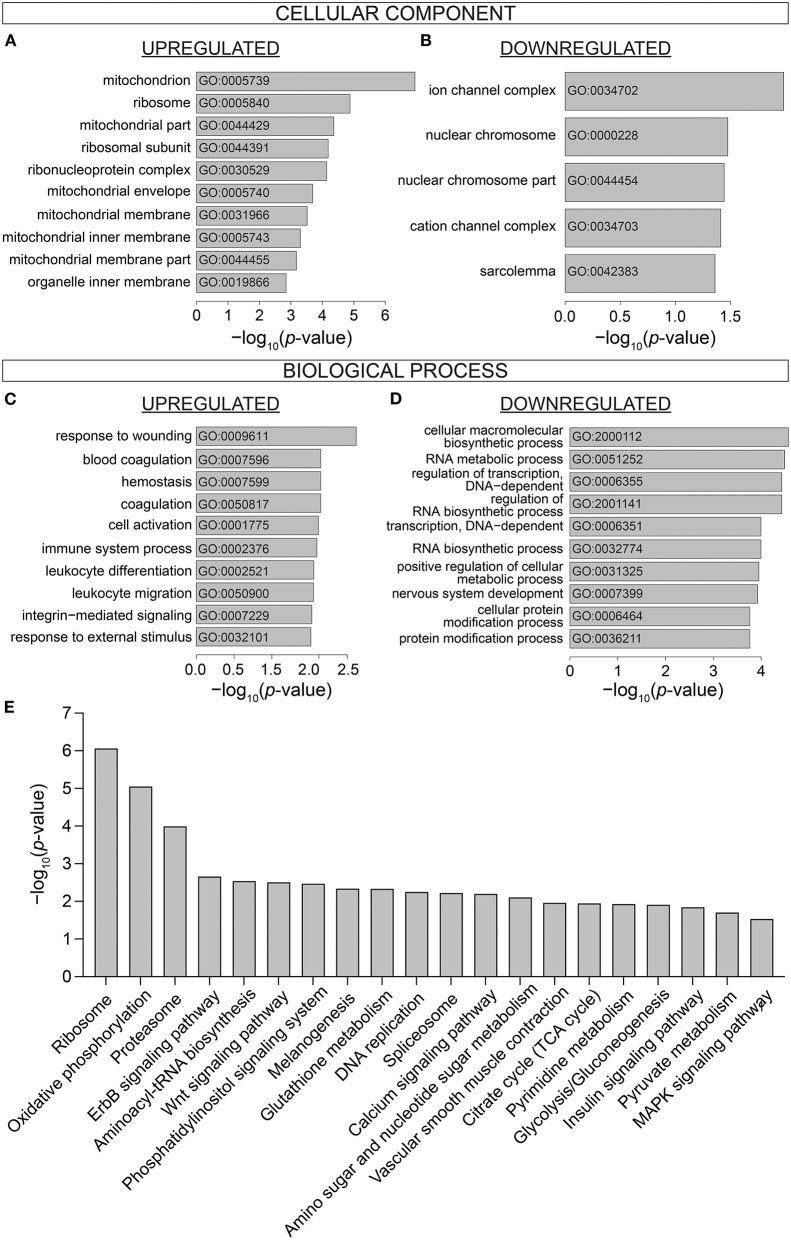
Gene Ontology and Pathway analysis of differentially expressed genes. Gene Ontology enrichment of **(A)** Upregulated and **(B)** Downregulated Cellular Component, **(C)** Upregulated and **(D)** Downregulated Biological Process. **(E)** KEGG Pathway analysis showing the top 20 most significantly differentially affected pathways in iron-deficiency anemia. *n* = 9 mice per group.

To investigate the biological processes affected by IDA, Gene Ontology (GO) enrichment analysis was performed on DE genes using the topGo Bioconductor package (39). Enriched GO terms were compiled in terms of *Cellular Component* and *Biological Process*. Cellular Components most affected by IDA included components of the mitochondria (GO:0005739, GO:0044429, GO:0005740, GO:0031966, GO:0005743, GO:0044455), the ribosome (GO:0005840, GO:0044391, GO:0030529) (Figure 3A), ion channels (GO:0034702, GO:0034703), and nuclear compartment (GO:0000228, GO0044454) (Figure 3B). Enriched Biological Processes related to stress (GO:0009611, GO: 0032101), immunity (GO:002376, GO:0002521, GO:0050900) (Figure 3C), transcriptional processes (GO:0006355, GO:0006351), macromolecule biosynthetic processes (GO:2001141, GO:0032774), and protein modification processes (GO:0006464, GO:0036211) (Figure 3D).

DE genes were further analyzed in terms of differentially regulated pathways. KEGG pathways were identified by Generally Applicable Gene Set Enrichment (GAGE) (Figure 3E). Top differentially regulated pathways included various processes associated with genomic and proteomic regulation and synthesis (Ribosome, mmu03010; Proteasome, mmu03050; Aminoacyl-tRNA biosynthesis, mmu00970; DNA replication mmu03030; Splicesome, mmu03040; Amino sugar and nucleotide sugar metabolism, mmu00520). Signaling pathways were also differentially regulated in the hearts of IDA mice (ErbB, mmu04012; Wnt, mmu04310; Phosphatidylinositol, mmu04070; MAPK, mmu04010; Insulin, mmu04910). Processes related to cardiac contractile function (Calcium signaling pathway, mmu04020; vascular smooth muscle contraction, mu04270) were also differentially affected in IDA.

### Iron-Deficiency Anemia Affects Metabolic Pathways in the Heart

Notably, numerous processes that regulate cardiac metabolism and energetics were highly enriched in IDA mice. The most affected Cellular Components were the mitochondria (Figure 3A). Pathways such as Oxidative phosphorylation (mmu00190), Glycolysis/gluconeogenesis (mmu00010), Pyruvate metabolism (mmu00620), and Citrate cycle (TCA cycle; mmu00020) (Figure 3E) were also among those that were most differentially affected in IDA. The individual components within the metabolic pathway affected in IDA are visualized in the pathway diagram shown in Supplementary Figure 2.

### Iron-Deficiency Anemia Affects Cardiac Complexes I and IV of Electron Transport Chain

Alterations in metabolic pathways are a common signature in heart disease (40–42). In the case of hypoxia, it is well-established that insufficient oxygen supply prompts genetic and cellular reprogramming that actively lowers mitochondrial oxidative phosphorylation (OXPHOS) and instead promotes glycolysis as an alternative pathway for ATP production (43, 44). To determine whether the genetically altered metabolic pathways, identified by RNA-seq, were also altered at the functional level, the metabolic state of iron-deficient and anemic mice was further investigated both *in vitro* and *in vivo*.

**Figure 4:**
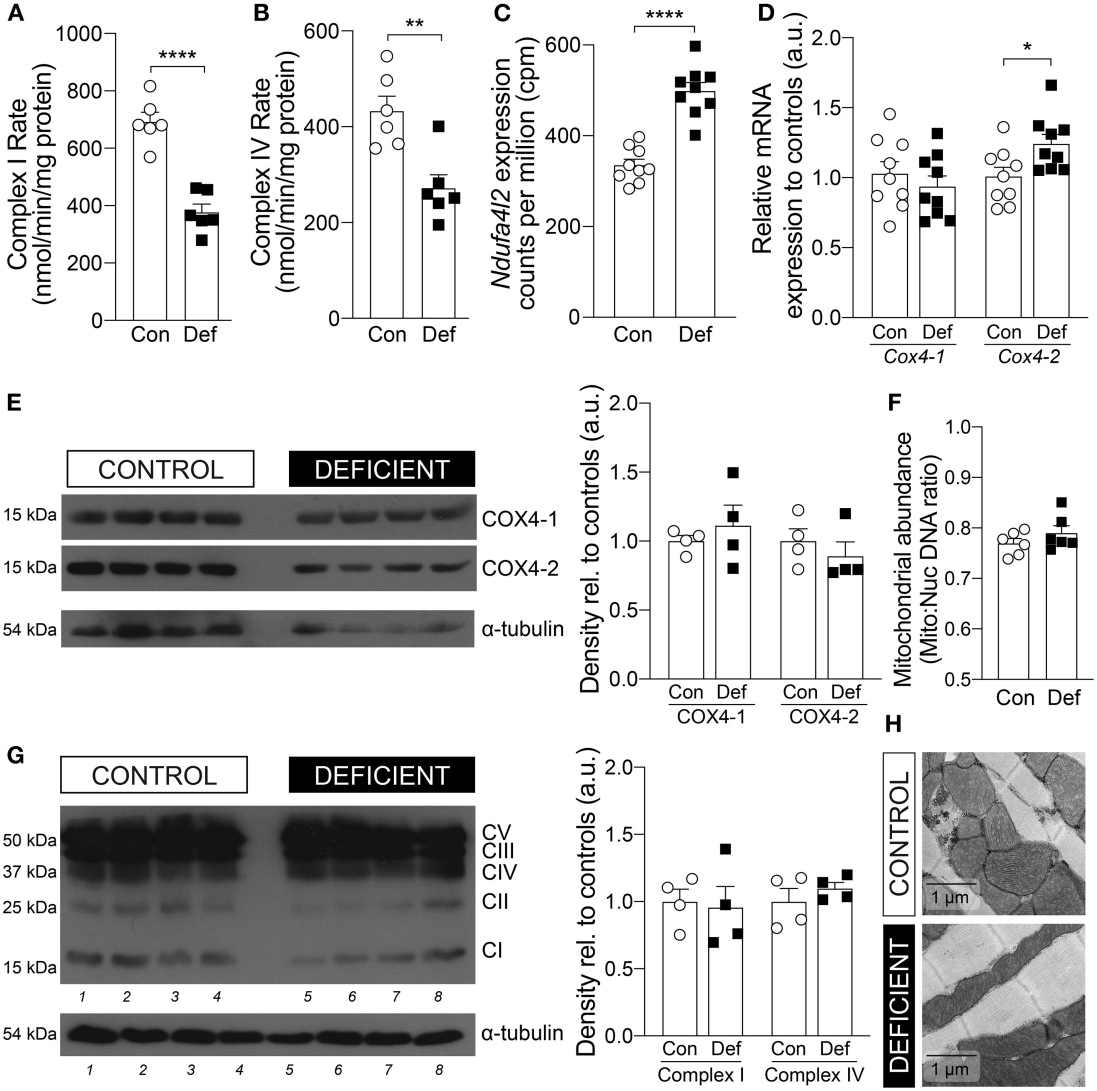
Cardiac mitochondrial OXPHOS activity and expression. Enzymatic activities of **(A)** Complex I and **(B)** Complex IV in hearts from mice on a control (Con) or iron-deficient (Def) diet. *n* = 6 mice per group. **(C)** mRNA expression level of *Ndufa4l2* measured by RNA-seq. *n* = 9 mice per group. **(D)** mRNA expression level of *Cox4-1* and *Cox4-2*. *n* = 9 mice per group. **(E)** Immunoblot of COX4-1 and COX4-2 protein expression in the heart and calculated density, normalized to α-tubulin loading control and relative to controls. *n* = 4 mice per group. **(F)** Mitochondrial abundance measured by comparing mitochondrial (mito) DNA to nuclear (nuc) DNA. *n* = 6 mice per group. **(G)** Protein expression of OXPHOS complexes and calculated density of Complex I and Complex IV, normalized to α-tubulin loading control and relative to controls. *n* = 4 mice per group. **(H)** Representative EM micrographs of cardiomyocytes from mice on a control or iron-deficient diet. Scale bar = 1 μm. All values plotted as mean ± sem. *P* values determined by unpaired, two-tail student's *t*-test. *p < 0.05, **p < 0.01, ****p < 0.0001.

First, to explore whether iron-deficiency anemia affects cardiac OXPHOS, the activities of the electron transport chain (ETC) complexes were measured in cardiac lysates obtained from hearts of IDA and control animals (Figure 4A). The activity of Complex I was significantly reduced by 45% in the hearts of IDA mice [control: 692.1 ± 33.1 vs. IDA: 376.9 ± 28.53 (nmol/min/mg protein); *p* < 0.0001]. Previously, it had been reported that reduction of Complex I activity in hypoxia is mediated by up-regulation of the gene encoding NADH dehydrogenase 1 alpha subcomplex, 4-like 2 (*Ndufa4l2*), which downregulates oxygen consumption (45). In the hearts of IDA mice, *Ndufa4l2* was significantly upregulated relative to expression levels in control hearts (Figure 4C).

Mitochondrial respiration is also regulated by additional mechanisms that maximize respiratory efficiency under conditions of reduced oxygen availability. Complex IV of the ETC is typically considered the mitochondrial oxygen sensing complex due to its high affinity for oxygen and its activity is reduced in hypoxia (44, 46). In the hearts of IDA mice, the rate of cardiac Complex IV activity was significantly decreased by 37% [control: 432.6 ± 31.2 vs. IDA: 271.6 ± 28.4 (nmol/min/mg protein); *p* = 0.0034; Figure 4B]. A component of Complex IV, Cytochrome *c* oxidase subunit 4 (COX4), which exists in two isoforms, is responsive to hypoxia. Whereas, isoform 1 of COX4 (COX4-1) is ubiquitously expressed, isoform 2 (COX4-2) has been shown to be a target of HIF-1α and overexpressed in conditions of low oxygen (47, 48). COX4-2 is suggested to increase COX activity under hypoxic conditions and represents mitochondrial remodeling that optimizes the efficiency of respiration when oxygen supply is low (49). In the hearts from IDA mice, there was a significant 20% increase in the mRNA expression of *Cox4-2* and a 36% increase in *Cox4-2* to *Cox4-1* mRNA ratio compared to controls (Figure 4D). However, at the protein level, the COX4-2 to COX4-1 ratio was not significantly different (Figure 4E).

The reduction in the activities of Complex I and Complex IV were not a result of changes in protein abundance of Complex I and Complex IV (Figure 4F), in mitochondrial number (Figure 4G), nor mitochondrial morphology (Figure 4H).

### Iron-Deficiency Anemia Decreases PDH Flux and Enhances Glycolysis

**Figure 5:**
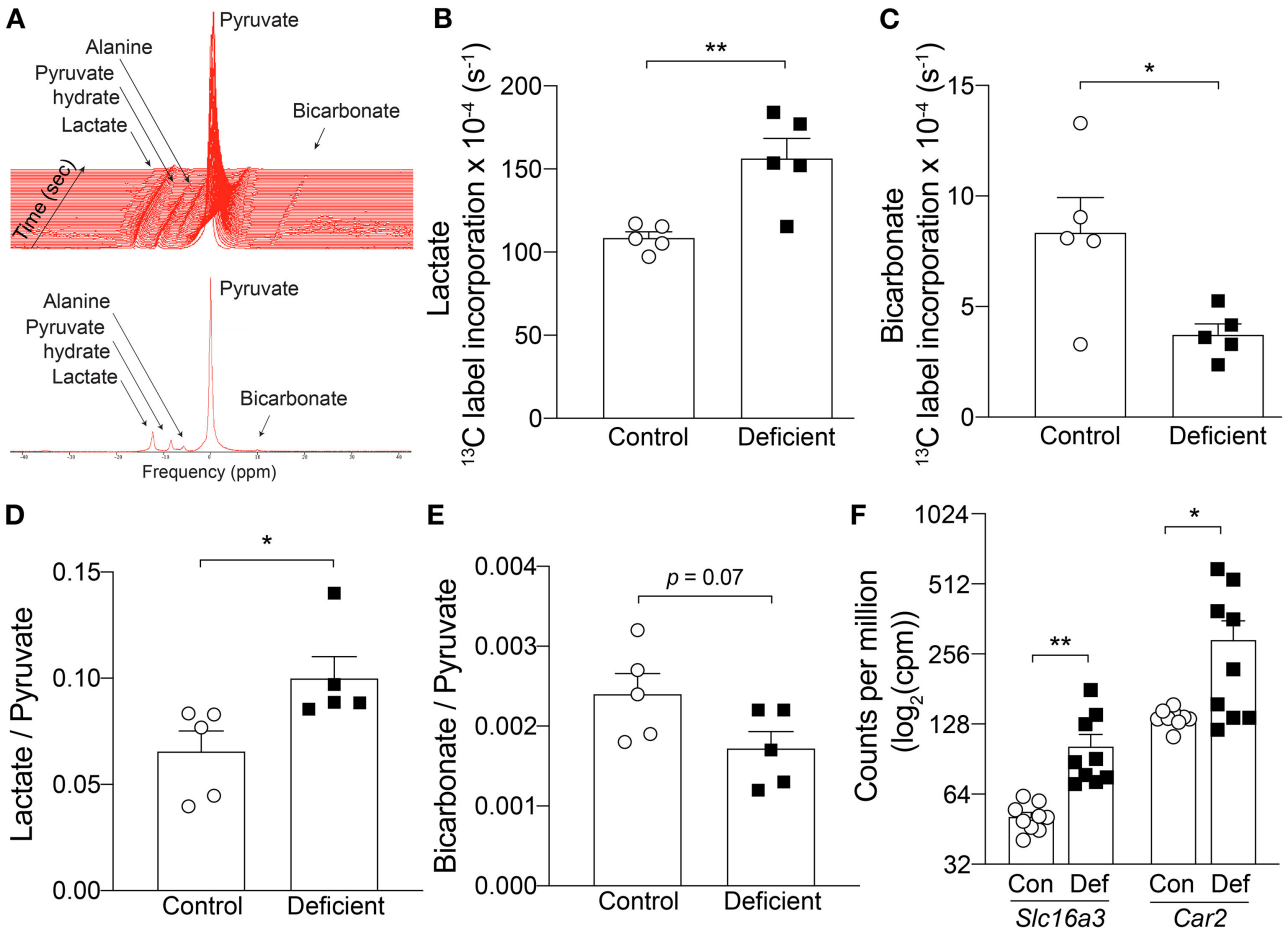
*In vivo* hyperpolarized [1-^13^C]pyruvate magnetic resonance spectroscopy in the heart. **(A)** Example spectra in a control mouse. Top, time course acquisition. Every line represents 1 sec. Bottom, Summed spectrum of 30 individual spectrum. ^13^C label incorporation kinetics into **(B)** Lactate and **(C)** Bicarbonate. *n* = 5 mice per group. **(D)** Lactate to pyruvate ratio, **(E)** Bicarbonate to pyruvate ratio. *n* = 5 mice per group. **(F)** mRNA expression level of *Slc16a3* and *Car2* measured by RNA-seq. *n* = 9 mice per group. Con, control; Def, Iron-deficient. All values plotted as mean ± sem. *P-*values determined by unpaired, two-tail student's *t*-test. *p < 0.05, **p < 0.01.

Impairment of oxidative metabolism is known to drive glycolysis as an alternative route of energy production (50, 51). Since OXPHOS was found to be impaired in the hearts of IDA mice, it is possible that metabolism is shifted toward glycolysis in these animals. To determine whether hearts of IDA mice manifested a shift from aerobic respiration to glycolysis, the cardiac metabolic state was investigated *in vivo* by following the metabolism of [1-^13^C]pyruvate using dynamic nuclear polarization (DNP)-hyperpolarized magnetic resonance spectroscopy (MRS). Pyruvate, the end-product of glycolysis, can be metabolized to either acetyl-CoA, lactate, or alanine (Figure 5A). Under aerobic conditions, pyruvate is decarboxylated into acetyl-CoA by the enzyme pyruvate dehydrogenase (PDH). CO_2_, the by-product of this process, is hydrated to bicarbonate. Because ^13^CO_2_ spectra are technically difficult to visualize, the rate of incorporation of ^13^C to bicarbonate is used to assess the flux through PDH (52). Under glycolytic conditions, pyruvate is preferentially converted to lactate by lactate dehydrogenase (53).

Two different methods were used to assess *in vivo* pyruvate metabolism from the hyperpolarized MRS data. First, the kinetics of ^13^C incorporation from pyruvate to lactate and bicarbonate was determined by fitting an average time course to the peak areas of each of the metabolites and normalizing to the corresponding pyruvate signal intensity to account for any differences in initial polarization level between experiments (Figures 5B,C). In control animals, the rate of ^13^C label transfer to lactate and bicarbonate was 108.6 ± 3.6 (× 10^−4^) s^−1^ and 8.34 ± 1.6 (× 10^−4^) s^−1^, respectively. IDA led to a significant 44% increase in ^13^C labeling of lactate [156.5 ± 12.1 (× 10^−4^) s^−1^; *p* = 0.005] and significant 55% decrease in ^13^C transfer to bicarbonate [3.74 ± 0.48 (× 10^−4^) s^−1^; *p* = 0.02]. This result demonstrates an increase in lactate flux associated with IDA.

In a second approach, the averaged maximum peak area of each metabolite over 30 sec of acquisition from the first appearance of pyruvate (30 individual spectra) were summed and normalized to the summed, peak area of pyruvate. This represents the ratio of total ^13^C incorporation to either lactate or bicarbonate relative to the amount of polarized pyruvate delivered in each particular experiment. Compared to controls, in IDA mice, the lactate to pyruvate ratio was significantly increased by 51% (control: 0.066 ± 0.02 vs. IDA: 0.100 ± 0.01; *p* = 0.03) and bicarbonate to pyruvate ratio was decreased by 30% (control: 0.0024 ± 0.0003 vs. IDA: 0.0017 ± 0.0002; *p* = 0.07; Figures 5D,E). Taken together, the kinetic and summed data indicate an increase in lactic acid production and decrease in PDH flux in the heart of IDA mice.

The significant changes in pyruvate metabolism to lactate and bicarbonate could be attributable to changes in the key metabolic enzymes that catalyze the respective reactions. Shown in Figure 1L, the mRNA levels of the enzyme lactate dehydrogenase A (*Ldha*) was significantly increased in the hearts of IDA mice compared to controls. Similarly, the expression of the cardiac pyruvate dehydrogenase kinase 1 (*Pdk1*), which inhibits PDH, was significantly upregulated in IDA.

An increase in lactate production would require increased efflux of lactate and H^+^ to facilitate the venting of lactic acid and prevent intracellular acidification. Lactate transport is mediated by monocarboxylate transporters (MCTs), of which MCT4 is a target of HIF-1α and its expression is canonically induced by hypoxia (54). In the hearts of IDA mice, mRNA levels of the MCT4 gene (*Slc16a3*) were overexpressed compared to controls (Figure 5F). The gene expression of carbonic anhydrase 2 (*Car2*), which catalyzes the reversible hydration of CO_2_ and is thought to augment the transport activity of MCT4 (55), was also significantly upregulated in IDA (Figure 5F).

## Discussion

The present study is the first *in vivo* demonstration of the effects of iron-deficiency anemia on cardiac energetics. Its most important finding is that IDA engenders a hypoxia-like glycolytic shift in cardiac metabolism. This finding is of significance because it describes a direct mechanism that can explain at least part of the detrimental consequences of IDA in heart failure patients. Until now, many of the detrimental effects of anemia in heart failure have been attributed to systemic effects, such as those affecting renal function, vascular resistance, and blood pressure (56), as well as on exercise capacity and skeletal muscle energetics (57).

It is known that under hypoxic conditions, numerous changes occur to the OXPHOS machinery, many of which are mediated by HIF-α. Activation of HIF-1α induces expression of PDK1 (43, 58), which inhibits PDH, thereby limiting substrate availability for the citrate (TCA) cycle. HIF-α can also alter the composition of Complex IV, by increasing the degradation of COX4-1, which optimizes the Complex's activity under aerobic conditions and increasing the expression of COX4-2 isoform, which optimizes its activity under hypoxic conditions (45, 59).

In the mouse model used in this study, 6 weeks of restriction of dietary iron resulted not only in systemic iron deficiency (Figures 1A–F,I) but also in cardiac hypoxia evidenced by the induction of HIF-1α expression (Figure 1L). Iron deficiency, independent of anemia, could also raise a HIF-α mediated hypoxic response (32, 60). However, evidence for a change in cardiac iron metabolism was not apparent in IDA. Whereas, the expression of cardiac *Tfrc* was significantly increased in IDA, the mRNA that encodes for DMT1 (*Slc11a2*) was not affected (Supplementary Figure 1). In addition, the FPN gene *Slc40a1* expression was downregulated in the hearts of IDA mice, as would be expected in iron deficiency, but the expression of the hepcidin gene *Hamp* was unaffected (Figure 1I). The lack of a coordinated hepcidin/FPN response to minimize iron extrusion from the cardiac tissue suggests that cellular iron availability in the heart remains unaltered, despite the systemic iron deficiency. In support of this assertion, the protein expression and localization of FPN was unaffected by IDA (Figure 1J) and cardiac iron content in IDA mice remained unchanged [Figure 1G, (38)]. Therefore, although a redistribution of iron from labile and stored iron pool within the cardiac tissue cannot be completely excluded, the HIF-1α induction observed in the hearts of IDA mice is more likely to be a consequence of tissue hypoxia secondary to anemia than from iron deficiency in the cardiac tissue.

Consistent with a metabolic phenotype in hypoxia, hearts of IDA mice exhibited a switch from aerobic OXPHOS to glycolytic metabolism. Specifically, the activities of Complexes I and IV of the ETC were significantly reduced (Figures 4A,B), a metabolic remodeling aimed at lowering mitochondrial oxygen consumption. The decrease in Complex I activity was accompanied by a significant increase in the expression of the Complex I component *Ndufa4l2* (Figure 4C), recently shown to be a target of HIF-1α and an inhibitor of Complex I (45, 61). Furthermore, the expression of *Cox4-2* transcript was significantly induced in the hearts of anemic mice (Figure 4D) but at the protein level, COX4-2 expression was not significantly different to controls (Figure 4E). This may be due to a delay in changes at the transcript level to be reflected at the protein level, and may explain the reduction in the enzymatic activity of Complex IV in IDA (Figure 4B).

Metabolic remodeling in anemia was further observed *in vivo* using hyperpolarized [1-^13^C]pyruvate MRS imaging. Along with the reduction in OXPHOS activity, the hearts of anemic mice exhibited significant reduction in PDH flux (Figures 5B,E) accompanied by an increase in *Pdk1* expression (Figure 1L). Instead, production of lactate was significantly increased (Figures 5A,D), along with a significant increase in the expression of *Ldha* (Figure 1L). These physiological changes in cardiac metabolism were consistent with alterations observed at the transcriptomic level. IDA resulted in significant changes to components of the mitochondria (Figure 3A) and affected the cardiac metabolic pathways *Oxidative phosphorylation, Pyruvate metabolism*, and *Glucose metabolism* (Figure 3E).

A shift toward a glycolytic phenotype would require increased efflux of lactic acid to prevent H^+^ accumulation and acidification of pH_i_. The mRNA expression of the lactate transporter MCT4 and its facilitator CA2 were significantly increased in the hearts of IDA mice. Despite these changes, resting pH_i_ of cardiomyocytes from IDA mice was found to be significantly reduced, as a result of decreased activity of the Na^+^/H^+^ exchanger-1 (NHE1), a major acid extruder in cardiomyocytes (62). A reduction in pH_i_ would affect cardiac contractility, which, in IDA mice, has been previously shown (38).

### Iron-Deficiency Anemia Induces Stress Response in the Heart

Metabolic remodeling was not the only gene expression signature of anemic hearts. Remarkably, IDA resulted in considerable global alterations to the cardiac transcriptome, with no overlapping gene signature with control hearts (Figures 2A,B) and over 1,500 differentially expressed genes (Figure 2C). One of the most affected pathways in IDA was that related to *stress response* (Figure 3). Adaptation to stress involves an extensive reorganization of gene expression and is achieved, in part, by regulation of mRNA biogenesis and mRNA fate (63). It is also well-known that both DNA replication and protein synthesis are significantly affected under hypoxic conditions (64). In the hearts obtained from IDA mice, which showed signs of hypoxia (Figures 1K,L), *Ribosome, DNA replication*, and *Proteosome* were among the most differentially regulated pathways (Figure 3E). Also, processes involved in transcription, various RNA-dependent processes, and nuclear chromosome were among the most enriched GO terms (Figures 3B,D). Nucleosome remodeling is considered an important component in stress-induced changes to gene expression, as it allows the transcription machinery access to stress-responsive genes (63).

Another mechanism of stress response involves modulation of cellular function via signal transduction (63). Among the most differentially affected signaling pathways were Phosphatidylinositol signaling, ErbB signaling, Wnt signaling, MAPK signaling in the hearts of IDA mice. Although the exact physiological consequences of these changes require further studies, gene expression changes constitute a major part of the physiological response to stress, and taken together, this pattern of differential gene enrichment is consistent with an adaptive stress response.

### Iron-Deficiency Anemia Affects Cardiac Contraction Pathway

Interestingly, Gene Ontology analysis also resulted in enrichment of the cellular components *ion channel complex, sarcolemma*, and *cation channel complex* (Figure 3B). These ontologies are represented by genes such as voltage-gated calcium channels, ryanodine receptors (RyR2), and sodium and potassium channels, all of which are critical components of cardiac contraction (65) and action potential (66), respectively. In particular, *Calcium signaling* was identified among the most significantly differentially regulated pathways. Previous studies have observed a decrease in cardiac contraction, both *in vivo* and *in vitro*, via downregulation of RyR2 channels and the sarco/endoplasmic reticulum Ca^2+^-ATPase (SERCA) pump activity in iron-deficiency anemia (38).

The deleterious effects of anemia on cardiac health is well-documented, but the mechanisms by which it directly affects cardiac physiology has not yet been ascertained. Using a non-candidate approach, we show, for the first time, that iron-deficiency anemia affects cardiac metabolism both at the transcriptional and physiological level in a manner akin to adaptation to chronic hypoxia. A shift toward a glycolytic metabolism is expected to lower ATP availability and therefore likely cause disruption in normal cardiac processes, such as contractility, which is a major ATP consuming pathway in the cardiac myocyte.

## Data Availability Statement

The RNA-seq data presented in this study can be found in the GEO online repository, accession number GSE162493.

## Ethics Statement

The animal study was reviewed and approved by Animal Welfare and Ethical Review Body, University of Oxford.

## Author Contributions

YJC, MKC, and VB performed experiments. YJC and PS analyzed data and wrote the paper. YJC, SLL, and PAR designed research. All authors contributed to the article and approved the submitted version.

## Conflict of Interest

The authors declare that the research was conducted in the absence of any commercial or financial relationships that could be construed as a potential conflict of interest.
