# Supplementary material for: Iron-Deficiency Anemia Results in Transcriptional and Metabolic Remodeling in the Heart Toward a Glycolytic Phenotype
Source: Front Cardiovasc Med. 2021 Jan 21;7:616920. doi: 10.3389/fcvm.2020.616920 (PMC7859254; doi:10.3389/fcvm.2020.616920)
Supplement: Supplementary file 1 [file Data_Sheet_1.docx]

SUPPLEMENTARY FIGURES

Iron-deficiency anemia results in transcriptional and metabolic remodeling in the heart toward a glycolytic phenotype

Short title: Cardiac transcriptome in iron-deficiency anemia

Yu Jin CHUNG^1,2*^, Pawel SWIETACH^1^, M. Kate CURTIS^1^, Vicky BALL^1^, Peter A. ROBBINS^1^, Samira LAKHAL-LITTLETON^1^.

*(1) Department of Physiology, Anatomy and Genetics, University of Oxford, Oxford, United Kingdom*

**Correspondence:**

Yu Jin Chung

[yujin.chung@kcl.ac.uk](mailto:yujin.chung@kcl.ac.uk)

*(2)* The Rayne Institute, 4^th^ Floor, Lambeth Wing, St Thomas’ Hospital, London SE1 7EH, UK. Telephone: +44 (0)20 7188 9613

Figure number: 2

**Figure S1. Expression of 3’ IRE-containing mRNA transcripts in iron-deficiency anemia. (A)** *Tfrc* encoding for the transferrin receptor (TFRC) protein. **(B)** *Slc11a2* encoding for the divalent metal transpoter-1 (DMT1) protein. Expression assessed by RNA-sequencing. *n* = 9 mice per group. All values plotted as mean ± sem. *P* values determined by unpaired, two-tail student’s *t*-test. * *p*<0.05.

**Figure S2. Iron-deficiency anemia affects mitochondrial oxidative phosphorylation pathway.** Visualization of differentially expressed genes affecting **(A)** the OXPHOS pathway and **(B)** Pyruvate metabolism, including glycolysis. Schematic rendered by the Bioconductor package PathView. Green = downregulated, Red = upregulated.
